# Bacterial etiology and antimicrobial resistance in bloodstream infections at the University of Gondar Comprehensive Specialized Hospital: a cross-sectional study

**DOI:** 10.3389/fmicb.2025.1518051

**Published:** 2025-03-13

**Authors:** Teshiwal Deress, Gizeaddis Belay, Getahun Ayenew, Worku Ferede, Minichil Worku, Tigist Feleke, Solomon Belay, Meseret Mulu, Asefa Adimasu Taddese, Tegegne Eshetu, Mebratu Tamir, Michael Getie

**Affiliations:** ^1^Department of Quality Assurance and Laboratory Management, School of Biomedical and Laboratory Science, College of Medicine and Health Sciences, University of Gondar, Gondar, Ethiopia; ^2^Department of Medical Microbiology, Amhara National Regional State Public Health Institute, Bahir Dar, Ethiopia; ^3^Department of Molecular Laboratory, Trachoma Elimination Program, The Carter Center, Bahir Dar, Ethiopia; ^4^Microbiology Laboratory, University of Gondar Comprehensive Specialized Hospital, Gondar, Ethiopia; ^5^Academy of Wellness and Human Development, Faculty of Arts and Social Sciences, Hong Kong Baptist University, Hong Kong SAR, China; ^6^Department of Medical Parasitology, School of Biomedical and Laboratory Science, College of Medicine and Health Sciences, University of Gondar, Gondar, Ethiopia

**Keywords:** bacterial etiology, antimicrobial resistance, bloodstream infections, University of Gondar Comprehensive Specialized Hospital, cross-sectional study, Ethiopia

## Abstract

**Background:**

Bacterial bloodstream infections are a major global health concern, particularly in resource-limited settings including Ethiopia. There is a lack of updated and comprehensive data that integrates microbiological data and clinical findings. Therefore, this study aimed to characterize bacterial profiles, antimicrobial susceptibility, and associated factors in patients suspected of bloodstream infections at the University of Gondar Comprehensive Specialized Hospital.

**Methods:**

A cross-sectional study analyzed electronic records from January 2019 to December 2021. Sociodemographic, clinical, and blood culture data were analyzed. Descriptive statistics and binary logistic regression were employed to identify factors associated with bloodstream infections. Descriptive statistics such as frequency and percentage were computed. Furthermore, a binary and multivariable logistic regression model was fitted to determine the relationship between BSI and associated factors. Variables with *p*-values of <0.05 from the multivariable logistic regression were used to show the presence of statistically significant associations.

**Results:**

A total of 4,727 patients’ records were included in the study. Among these, 14.8% (701/4,727) were bacterial bloodstream infections, with Gram-negative bacteria accounting for 63.5% (445/701) of cases. The most common bacteria were *Klebsiella pneumoniae* (29.0%), *Staphylococcus aureus* (23.5%), and *Escherichia coli* (8.4%). The study revealed a high resistance level to several antibiotics, with approximately 60.9% of the isolates demonstrating multidrug resistance. *Klebsiella oxytoca*, *Klebsiella pneumoniae*, and *Escherichia coli* exhibited high levels of multidrug resistance. The study identified emergency OPD [AOR = 3.2; (95% CI: 1.50–6.74)], oncology ward [AOR = 3.0; (95% CI: 1.21–7.17)], and surgical ward [AOR = 3.3; (95% CI: 1.27–8.43)] as factors associated with increased susceptibility to bloodstream infections.

**Conclusion:**

The overall prevalence of bacterial isolates was high with concerning levels of multi-drug resistance. The study identified significant associations between bloodstream infections with age groups and presentation in specific clinical settings, such as the emergency OPD, oncology ward, and surgical ward. Strict regulation of antibiotic stewardship and the implementation of effective infection control programs should be enforced.

## Background

Bacterial bloodstream infections (BSIs) are a significant global concern (Matakone et al., 2024). These infections can be acquired in hospitals or within the community. Microorganisms typically enter the bloodstream through lymphatic drainage from local lesions or direct invasion of blood vessels (Schamroth et al., 2024; Murray et al., 2022). Clinically, BSIs manifest through various symptoms, including fever, disseminated intravascular coagulation, hypotension, hyperventilation, excessive sweating, endocarditis, and septic shock. Blood culture remains the gold standard for diagnosing BSIs (Misha et al., 2021; Belew et al., 2023; An et al., 2023).

Pathogenic bacteria are the primary contributors to most bloodstream infections (Karuna et al., 2023). Among the Gram-negative bacteria commonly isolated from BSI patients are *Klebsiella pneumoniae*, *Acinetobacter baumannii, Escherichia coli*, *Pseudomonas aeruginosa, Neisseria meningitidis*, and *Haemophilus influenzae.* Gram-positive bacteria frequently identified include *Staphylococcus aureus*, *Streptococcus pneumoniae*, *Streptococcus pyogeneses*, *Streptococcus agalactiae*, and *Enterococcus faecium* (Foglia et al., 2023). Occasionally, fungal pathogens are also detected in BSI patients (Diekema et al., 2000). In 2019, BSIs were responsible for over 2 million deaths worldwide (Ikuta et al., 2022). In Ethiopia, the prevalence of BSIs ranges from 12.84 to 27.78% across various studies (Tsegaye et al., 2021; Birru et al., 2021). Effective management of BSIs involves immunization programs, early detection, swift microbiological diagnosis, and rapid initiation of appropriate antimicrobials, aligning with WHO control strategies (Collignon et al., 2016). Antimicrobials play a crucial role in managing BSIs by eliminating or inhibiting microbial proliferation (Bashir et al., 2023).

While active antimicrobial therapy remains the cornerstone of BSI management, the escalating emergence of drug-resistant bacteria significantly exacerbates morbidity and mortality (Matakone et al., 2024; Foglia et al., 2023; Schöneweck et al., 2021). Crucially, evidence indicates that infections caused by antimicrobial-resistant (AMR) pathogens result in a two to threefold increase in various negative outcomes. These include increased disease severity, hospital admission rates, length of hospitalization, healthcare costs, morbidity, and mortality, compared to infections caused by susceptible strains (Minton et al., 2008; Aidara-Kane et al., 2018). The bacterial epidemiology and AMR profiles in BSI patients exhibit considerable heterogeneity across different settings, due to geographical location, pathogen strains, underlying genetic variations, and access to antimicrobial agents (Bhattarai et al., 2021; Diekema et al., 2019; Legese et al., 2022; Niederman et al., 2021). The situation in Ethiopia is particularly concerning, where both Gram-positive and Gram-negative bacteria demonstrate significant resistance to commonly used first-and second-line antibiotics, such as ampicillin, gentamicin, and third-generation cephalosporins (Sorsa et al., 2019; Adane et al., 2020; Abebe et al., 2021). Furthermore, studies conducted in Ethiopia have reported a high prevalence of multidrug-resistant (MDR) isolates, ranging from 47 to 72% (Sorsa et al., 2019; Gebremariam et al., 2022).

Although existing studies have investigated bacterial etiology and resistance profiles in BSIs, AMR remains a dynamic and escalating threat to global public health. This study aims to provide a more comprehensive perspective by incorporating clinical findings to correlate pathogen profiles with patient outcomes, an aspect often underreported in previous research. It also provides updated data on bacterial pathogens and their susceptibility patterns. Given that AMR rates vary over time and by region, this study’s updated data will be vital for tracking regional trends and adjusting treatment strategies accordingly. This study seeks to fill a critical knowledge gap, improve patient outcomes, and enhance understanding of BSIs in the region. Therefore, this study aimed to characterize bacterial pathogens, antimicrobial susceptibility profiles, and associated factors among BSI patients at the University of Gondar Comprehensive Specialized Hospital (UoGCSH), Ethiopia.

## Methods

### Study setting

The study was conducted at the UoGCSH, in central Gondar town, 747 km northwest of Addis Ababa (the capital city of Ethiopia). This hospital serves as a referral center, serving a large population of over seven million individuals residing in its catchment area and surrounding regions (Gobezie et al., 2023). It provides a wide range of diagnostic and treatment services across various medical disciplines. The hospital’s laboratory is organized into specialized sections, including dedicated units for clinical chemistry, hematology, parasitology, *Mycobacterium tuberculosis* culture, molecular biology, serology, viral load, and microbiology. It also has one main laboratory. The microbiology laboratory plays a vital role in bacteriological diagnostics. This laboratory is equipped to perform a range of procedures, including Gram staining, conventional culture techniques, biochemical tests, and antimicrobial susceptibility tests. The data generated within this laboratory is documented, serving dual purposes: direct patient care management and contributing to broader research initiatives.

### Study design and period

An institution-based cross-sectional study was conducted retrospectively from January 2019 to December 2021 at the UoGCSH. The primary objective was to identify and characterize bacterial pathogens responsible for bloodstream infections and evaluate their antimicrobial susceptibility patterns. Blood samples were collected from patients with suspected bloodstream infections. These samples underwent microbiological analysis to isolate and identify the bacterial pathogens. Following isolation, antibiotic susceptibility testing was performed on the identified bacteria to determine their resistance profiles to commonly used antibiotics.

### Population and sampling

Laboratory records of patients suspected of having BSIs were gathered from the electronic database. These records contain valuable information about demographic, clinical, and blood culture results of the specific bacterial isolates associated with the infections. The study employed a comprehensive sampling approach, including all available laboratory records of patients of all age groups suspected of having BSIs. The inclusion criteria were suspicion of BSIs; complete records including demographic variables, clinical diagnosis, and blood culture results; and presentation at any of the following UoGCSH units: adult inpatient, emergency triage assessment and treatment (ETAT), emergency OPD, gynecology ward, medical ICU, medical OPD, neonate ICU, oncology ward, pediatric ICU, pediatric inpatient, surgical OPD, and surgical ward.

### Antimicrobial susceptibility testing practices

Blood samples were collected and processed according to standard operating procedures. Before blood drawing, the selected venipuncture sites were thoroughly cleansed with 70% alcohol followed by 2% iodine tincture. Approximately 8–10 mL of blood is routinely collected from adults, 1–4 mL from children, and 1 mL from neonates, obtained in two draws taken 30 min apart. Blood samples are then transferred to broth containing 0.025% sodium polyanetholsulfonate at a 1:10 ratio and incubated aerobically at 35–37°C for 5 days. During this 5-day incubation period, the broth media are monitored daily for bacterial growth through visual examination. Broth cultures exhibiting signs of microbial growth are Gram-stained and subsequently sub-cultured onto blood agar, chocolate agar (either in a CO2 incubator or a candle jar), and MacConkey agar plates. These plates are incubated aerobically at 35–37°C for 18–24 h. Furthermore, after 18–24 h of incubation, a blind subculture is performed for all negative blood culture bottles by streaking onto chocolate agar plates. Bacterial pathogens are identified based on macroscopic colony characteristics, including color, size, shape, and texture, as well as Gram staining. Gram-negative bacterial pathogens are identified using a series of conventional biochemical tests, including indole, urease, lysine decarboxylase, triple sugar iron agar, citrate utilization, oxidase, and sulfide indole motility tests. Conversely, Gram-positive bacteria and their hemolytic patterns are identified based on catalase and coagulase tests (Arega et al., 2018; Arega et al., 2017).

### Antimicrobial susceptibility testing practices

Antimicrobial susceptibility testing was performed using the Kirby-Bauer disc diffusion method. Three to five pure colonies of bacteria were selected and transferred to a tube containing 4–5 mL of sterile normal saline. The mixture was gently mixed to form a homogenous suspension. The prepared suspension was incubated at 35–37°C until the turbidity matched a 0.5 McFarland standard. A sterile cotton swab was then dipped into the prepared suspension, and excess fluid was removed by pressing it against the inside wall of the test tube. The suspension was evenly distributed on the surface of Mueller-Hinton agar for non-fastidious bacteria and on Mueller-Hinton agar supplemented with 5% sheep blood for fastidious bacteria. The inoculated plates were left at room temperature for 3–15 min before placing the antibiotic discs. The common antimicrobial agents tested, chosen based on the Clinical Laboratory Standards Institute (CLSI) M100 guidelines 2019 (Clinical and Laboratory Standards Institute, 2019), included penicillin (10 μg), cefoxitin (30 μg), gentamicin (10 μg), ampicillin (10 μg), cefotaxime (30 μg), ceftriaxone (30 μg), vancomycin (30 μg), chloramphenicol (30 μg), tobramycin (10 μg), amoxicillin-clavulanate (20/10 μg), meropenem (10 μg), amikacin (30 μg), ciprofloxacin (5 μg), piperacillin-tazobactam (100/10 μg), trimethoprim-sulfamethoxazole (1.25/23.75 μg), and ceftazidime (30 μg). The plates were then incubated at 35–37°C for 16–18 h, after which the diameters of the zones of inhibition around the disks were measured. The degree of susceptibility of the bacterial isolates was then classified as sensitive, intermediate, or resistant based on the standardized table provided by the CLSI guidelines (Clinical and Laboratory Standards Institute, 2019).

### Data extraction

Data were extracted from the electronic health records of the UoGCSH microbiology laboratory by two experienced clinical laboratory professionals. The extraction checklist included patients’ demographic information, such as gender and age, clinical setting (Ward, OPD, ICU, etc.), and admission date. It also captured information on clinical presentations or indications, including fever, hypothermia, hypotension, increased respiratory rate, confusion, suspected pneumonia, indications of complicated urinary tract infection, suspected meningitis, suspected abscess, osteomyelitis, suspected soft tissue infection, suspected abdominal infection, severe malaria, typhoid fever, neonatal infection, indications of other severe infections, and any other relevant indications. Furthermore, the checklist encompassed information about antibiotic therapy administered within 2 weeks before sample collection for laboratory diagnosis, specifying the type of antibiotic used. Furthermore, data on culture media and microbial identification, antibiotic disks used, and susceptibility results were also collected. Bacterial isolates exhibiting intermediate resistance to antimicrobial agents were categorized as resistant strains.

### Operational and case definitions

Neonate: A neonate is defined as a newborn infant within the first 28 days of life (Bejitual et al., 2022).

Pediatrics: The pediatric population encompasses individuals from early infancy (above 28 days) through adolescence (up to 18 years).

Adult: Adults are defined as individuals aged 18 years and older.

### Age categorization

- Less than 1 month: This group represents a particularly vulnerable population with an immature immune system that relies on maternal antibodies.
- One month to less than 3 years: This age range encompasses infants and toddlers, during which there is rapid immune system development and increased exposure to environmental pathogens due to enhanced mobility and weaning.
- Three years and above: This group includes older children, adolescents, and adults, where the immune system is generally more mature.

Fever: A patient was defined as having a fever if their body temperature was 37.5°C to 38°C during admission. This measurement was documented by healthcare providers, alongside information gathered from patients and/or their caregivers regarding any fever episodes in the 48 h preceding admission (Egi and Morita, 2012; Mackowiak et al., 2021).

Hypothermia: Body temperature measured by a reliable method (axillary, oral, tympanic, or rectal) below 36.5°C for neonates (Alebachew Bayih et al., 2019), for pediatric 36.0°C (Nemeth et al., 2021; Rauch et al., 2021; Zhao et al., 2023), and for adults 36.0°C (Savioli et al., 2023; Haverkamp et al., 2018).

Hypotension: Defined based on age-specific systolic blood pressure (SBP) thresholds.

- Neonates (≤28 days): SBP < 60 mmHg (Khilnani et al., 2010)
- Infants (2 months to <1 year): SBP < 70 mmHg (Khilnani et al., 2010)
- Children (1 to <10 years): SBP <70 + (2 × 𝑎𝑔𝑒 in years) 70 + (2 × age in years) mmHg (Khilnani et al., 2010)
- Adolescents (≥10 to <18 years): SBP < 90 mmHg (Khilnani et al., 2010)
- For adults blood pressure systolic below BP < 90 (Khilnani et al., 2010; Sharma et al., 2025).

Multidrug resistance: This describes the capability of bacterial isolates to resist the effects of antimicrobial agents from three or more distinct categories or groups (Belew et al., 2023; Alam et al., 2011; Garoy et al., 2021; Bitew et al., 2023; Hailemariam et al., 2021).

### Data analysis and interpretation

Datasets were exported from electronic health records into Microsoft Excel spreadsheets for completeness checking and data cleaning. Subsequently, the cleaned data was transferred to STATA software (StataCorp. 2021. Stata Statistical Software: Release 17. College Station, TX: StataCorp LLC.) for coding and analysis. Descriptive statistics, including frequencies and percentages, were calculated. Furthermore, binary and multivariable logistic regression models were employed to investigate the relationship between BSI and associated factors. All variables exhibiting a *p*-value of ≤0.25 in the bivariable analysis were included in the multivariable logistic regression analysis. Variables with a *p*-value of <0.05 in the multivariable logistic regression analysis were considered to indicate statistically significant associations. The study findings are presented in textual descriptions, tables, and graphical formats.

### Quality control protocol

Blood sample collection, culture media preparation, inoculation, incubation, and biochemical tests were conducted according to standard operating procedures for microbiological techniques. The sterility of the culture media was checked by randomly selecting 5% of the prepared media, which was then incubated aerobically at 35–37°C for 24 h. Furthermore, all freshly prepared blood agar, MacConkey agar, and chocolate agar media were regularly checked by inoculating 5 % of them with known standard strains of *S. aureus* ATCC 25923, *S. epidermidis* ATCC 19211, *S. pneumoniae* ATCC 49619, *E. coli* ATCC 25922, *P. mirabilis* ATCC 12453, and *H. influenzae* ATCC 47112. Microbiology experts performed culture media inoculation, colony characterization, and antibiotic susceptibility tests. The authors developed a standardized data collection form containing variables of interest. The accuracy and completeness of the collected data were then assessed using techniques such as checking for missing values and conducting duplicate checks.

### Ethical consideration

Before conducting the study, ethical clearance was obtained from the University of Gondar, Institutional Review Board (Ref: VP/RTT/05/822/2024). Permission was also obtained from the hospital’s clinical director’s office and the head of the microbiology laboratory section. Patient details were removed to ensure confidentiality, and the data were analyzed anonymously. No personal identifiers were used, and the collected data was only accessible to the investigator.

## Results

### Socio-demographic characteristics of study participants

This study included 4,727 patients admitted to UoCGSH between January 2019 and December 2021 who were suspected of BSIs and provided blood samples for diagnosis. Of these patients, 58.1% (2,747/4,727) were male. The study population’s age ranged from 1 day to 96 years. Neonates represented 26.3% (1,244/4,727), pediatric patients 38.5% (1,821/4,727), and adult patients 35.2% (1,662/4,727) of the total participants (Table 1).

**Table 1:** Socio-demographic characteristics of patients suspected of bloodstream infections at UoGCSH, Ethiopia, 2024 (*n* = 4,727).

Variables	Category	Frequency	Percentage (%)
Gender	Male	2,747	58.1
	Female	1,980	41.9
Age	Less than 1 month	1,259	26.6
	From 1 month to less than 3 years	909	19.2
	3 years and above	2,559	54.1
Patient category	Neonate	1,244	26.3
	Pediatric	1,821	38.5
	Adult	1,662	35.2
Clinical setting	Adult inpatient	624	13.2
	ETAT	781	16.5
	Emergency OPD	51	1.1
	Gynecology ward	16	0.3
	Medical ICU	192	4.1
	Medical OPD	701	14.8
	Neonate ICU	1,246	26.4
	Oncology ward	73	1.5
	Pediatric ICU	562	11.9
	Pediatric inpatient	416	8.8
	Surgical OPD	36	0.8
	Surgical ward	29	0.6
Year	2019	1,110	23.5
	2020	1,611	34.1
	2021	2,005	42.4

ETAT, emergency triage assessment and treatment; ICU, intensive care unit; OPD, outpatient department.

### Clinical diagnosis of study participants

Fever was the predominant clinical presentation among patients suspected of BSI at the UoGCSH, observed in 87.6% of cases. A significant proportion of these patients (35.5%) had received antibiotic therapy within the 2 weeks preceding their admission. An elevated respiratory rate was noted in nearly a quarter of the study participants (23.6%). In contrast, several other clinical indicators were reported less frequently, including hypothermia (2.5%), signs of confusion (2.9%), a history of fever within the preceding 48 h (2.5%), and signs of hypotension (2.5%). Specific suspected sources of infection were generally infrequent, with pneumonia being the most prevalent at 13.4%. Other suspected sites, such as complicated urinary tract infections (1.6%), soft tissue infections (1.6%), and abdominal infections (1.5%), were less common. Notably, suspicion of neonatal infection was reported in 4.5% of the neonate subgroup (Table 2).

**Table 2:** Clinical diagnosis of bloodstream infection suspected patients at UoGCSH, Ethiopia, 2024 (n = 4,727).

Variables	Category	Patient category			Total n (%)
		Neonate *n* (%)	Pediatric *n* (%)	Adult *n* (%)	
Fever	Yes	1,008 (81.0)	1,633 (89.7)	1,503 (90.4)	4,144 (87.6%)
	No	236 (19.0)	188 (10.3)	159 (9.6)	583 (12.3%)
Increased respiratory rate	Yes	262 (21.1)	439 (24.1)	414 (24.9)	1,115 (23.6%)
	No	982 (78.9)	1,382 (75.9)	1,248 (75.1)	3,612 (76.4%)
Suspicion of neonatal infection	Yes	211 (0.4)	NA	NA	211 (4.5%)
	No	1,033 (83.0)	NA	NA	1,033 (21.9%)
Hypothermia	Yes	9 (0.7)	33 (1.8)	77 (4.6)	119 (2.5%)
	No	1,235 (99.3)	1,788 (98.2)	1,585 (95.4)	4,608 (97.5%)
Sign of confusion	Yes	10 (0.8)	48 (2.6)	80 (4.8)	138 (2.9%)
	No	1,234 (99.2)	1,773 (97.4)	1,582 (95.2)	4,589 (97.1%)
History of fever during the last 48 h	Yes	24 (1.9)	47 (2.6)	47 (2.8)	118 (2.5%)
	No	1,220 (98.1)	1,774 (97.4)	1,615 (97.2)	4,609 (97.5%)
Sign of hypotension	Yes	9 (0.7)	33 (1.8)	77 (4.6)	119 (2.5%)
	No	1,235 (99.3)	1,788 (98.2)	1,585 (95.4)	4,608 (97.5%)
Suspicion of complicated urinary tract infection	Yes	3 (0.2)	19 (1.0)	55 (3.3)	77 (1.6%)
	No	1,241 (99.8)	1,802 (99.0)	1,607 (96.7)	4,650 (98.4%)
Suspicion of pneumonia	Yes	111 (8.9)	216 (11.9)	307 (18.5)	634 (13.4%)
	No	1,133 (91.1)	1,605 (88.1)	1,355 (81.5)	4,093 (86.6%)
Suspicion of soft tissue infection	Yes	5 (0.4)	32 (1.8)	37 (2.2)	74 (1.6%)
	No	1,239 (99.6)	1,789 (98.2)	1,625 (97.8)	4,653 (98.4%)
Suspicion of abdominal infection	Yes	11 (0.9)	26 (1.4)	32 (1.9)	69 (1.5%)
	No	1,233 (99.1)	1,795 (98.6)	1,630 (98.1)	4,658 (98.5%)
Suspicion of abscess	Yes	4 (0.3)	33 (1.8)	30 (1.8)	67 (1.4%)
	No	1,240 (99.7)	1,788 (98.2)	1,632 (98.2)	4,660 (98.6%)
Suspicion of severe malaria	Yes	1 (0.1)	24 (1.3)	37 (2.2)	62 (1.3%)
	No	1,243 (99.9)	1,797 (98.7)	1,625 (97.8)	4,665 (98.7%)
Suspicion of osteomyelitis	Yes	2 (0.2)	13 (0.7)	7 (0.4)	22 (0.5%)
	No	1,242 (99.8)	1,808 (99.3)	1,655 (99.6)	4,705 (99.5%)
Suspicion of typhoid fever	Yes	0	4 (0.2)	9 (0.5)	13 (0.3%)
	No	1,244 (100.0)	1,817 (99.8)	1,653 (99.5)	4,714 (99.7%)
Suspicion of other severe infection	Yes	9 (0.7)	25 (1.4)	27 (1.6)	61 (1.3%)
	No	1,235 (99.3)	1,796 (98.6)	1,635 (98.4)	4,666 (98.7%)
Antibiotic therapy within 2 weeks before admission	Yes	408 (32.8)	690 (37.8)	582 (35.0)	1,680 (35.5%)
	No/unknown	836 (67.2)	1,131 (62.1)	1,080 (65.0)	3,047 (64.5%)
Type of antibiotic used before admission	Single	73 (5.9)	312 (17.1)	247 (14.9)	632 (13.4)
	Double	311 (25.0)	343 (18.8)	290 (17.5)	944 (20.0)
	Triple or more	24 (1.9)	35 (1.9)	45 (2.7)	104 (2.2)
	Not used	836 (67.2)	1,131 (62.1)	1,080 (65.0)	3,047 (64.4)

### Prevalence of bacterial isolates

Of the 4,727 blood specimens analyzed for microbial growth via blood culture, 17.5% (827) showed bacterial growth within the first day of incubation. After further incubation and subculture, the overall bacterial blood culture positivity rate was 14.8% (701 out of 4,727 specimens). These positive cultures comprised 63.5% Gram-negative bacteria (445 out of 701) and 36.5% Gram-positive bacteria (256 out of 701). BSIs were most frequently observed in neonates (40.8%, 286 out of 701) and pediatric patients (40.4%, 283 out of 701). The prevalence of bacterial isolates increased over the three-year study period: 3.8% in 2019, 4.4% in 2020, and 6.6% in 2021.

### Frequency distribution of bacterial isolates

A total of 27 distinct bacterial isolates were identified. *K. pneumoniae* was the most frequently isolated pathogen, accounting for 27.1% (203 out of 750) of all isolates, followed by *S. aureus* at 22.0% (165 out of 750). *S. viridans* was also prevalent, representing 6.5% (49/750) of the isolates. Non-fermenting Gram-Negative Rods (NFGNR) constituted 4.5% (34/750) of the isolates; the specific species could not be determined using routine biochemical methods. Among the identified Coagulase-Negative Staphylococci (CoNS), only 1.1% (8/701) were considered clinically significant in the diagnosis of endocarditis. The remaining CoNS isolates were likely contaminants and thus deemed clinically irrelevant. Other, less frequently isolated bacteria, each with a single isolation, included *Enterobacter aerogenes*, *P. multocida*, *S. arizonae, S. typhi,* and *Serratia* spp. (Table 3).

**Table 3:** Distribution of microbial isolates among gender and patient categories at the UoGCSH, Ethiopia, 2024 (*n* = 701).

Bacterial isolates	Frequency *n* (%)	Gender category		Age category		
		Male *n* (%)	Female *n* (%)	Neonate *n* (%)	Pediatric *n* (%)	Adult *n* (%)
*K. pneumoniae*	203 (29.0)	124 (61.1)	79 (38.9)	122 (60.1)	63 (31.0)	18 (8.9)
*S. aureus*	165 (23.5)	94 (57.0)	71 (43.0)	41 (24.8)	88 (53.3)	36 (21.8)
*E. coli*	59 (8.4)	42 (71.2)	17 (28.8)	15 (25.4)	22 (37.3)	22 (37.3)
*S. viridians*	49 (7.0)	31 (63.3)	18 (36.7)	16 (32.7)	24 (49.0)	9 (18.4)
*A. baumannii*	48 (6.8)	31 (64.6)	17 (35.4)	20 (41.7)	13 (27.1)	15 (31.3)
*E. cloacae*	44 (6.3)	28 (63.6)	16 (36.4)	23 (52.3)	14 (31.8)	7 (15.9)
*NFGNR*	34 (4.9)	17 (50.0)	17 (50.0)	14 (41.2)	12 (35.3)	8 (23.5)
*K. ozaenae*	17 (2.4)	9 (52.9)	8 (47.1)	4 (23.5)	11 (64.7)	2 (11.8)
*S. pneumoniae*	11 (1.6)	5 (45.5)	6 (54.5)	3 (27.3)	6 (54.5)	2 (18.2)
*Pseudomonas* spp.	10 (1.4)	7 (70.0)	3 (30.0)	3 (30.0)	4 (40.0)	3 (30.0)
*CoNS*	8 (1.1)	5 (62.5)	3 (37.5)	2 (25.0)	4 (50.0)	2 (25.0)
*Enterococcus* spp.	7 (1.0)	4 (57.1)	3 (42.9)	4 (57.1)	3 (42.9)	0
*K. oxytoca*	7 (1.0)	4 (57.1)	3 (42.9)	2 (28.6)	4 (57.1)	1 (14.3)
*Citrobacter* spp.	6 (0.9)	3 (50.0)	3 (50.0)	1 (16.7)	5 (83.3)	0
*S. agalactiaee*	6 (0.9)	3 (50.0)	3 (50.0)	2 (33.3)	3 (50.0)	1 (16.7)
*N. meningitidis*	5 (0.7)	3 (60.0)	2 (40.0)	1 (20.0)	2 (40.0)	2 (40.0)
*S. pyogenes*	5 (0.7)	3 (60.0)	2 (40.0)	1 (20.0)	3 (60.0)	1 (20.0)
*Providencia* spp.	4 (0.6)	3 (75.0)	1 (25.0)	2 (50.0)	0	2 (50.0)
*P. mirabilis*	3 (0.4)	2 (66.7)	1 (100)	1 (33.3)	1 (33.3)	1 (33.3)
*Salmonella group A*	3 (0.4)	1 (33.3)	2 (66.7)	2 (66.7)	1 (33.3)	0
*K. rhinoscleromatis*	2 (0.3)	1 (50)	1 (50)	2 (100)	0	0
*S. typhi*	1 (0.1)	1 (100)	0	1 (100)	0	0
*Seratia* spp.	1 (0.1)	1 (100)	0	1 (100)	0	0
*S. arizonae*	1 (0.1)	0	1 (100)	1 (100)	0	0
*P. multocida*	1 (0.1)	1 (100)	0	1 (100)	0	0
*E. aerogenes*	1 (0.1)	0	1 (100)	1 (100)	0	0
Total	701 (100)	423 (60.3)	278 (39.7)	286 (40.8)	283 (40.4)	132 (18.8)

### Antimicrobial susceptibility test results of bacterial isolates

Antimicrobial susceptibility test results of bacterial isolates showed significant variations in the susceptibility of Gram-positive bacteria. Specifically, CoNS isolates demonstrated 100% susceptibility to penicillin. In contrast, *S. pneumoniae* isolates showed only 20% susceptibility to vancomycin. *S. aureus* isolates, on the other hand, were predominantly resistant to both penicillin (87.6%) and oxacillin (61.1%). Comparatively, *Enterococcus* spp. isolates showed a significant level of resistance to vancomycin (57.1%) (Tables 4).

**Table 4:** Antimicrobial susceptibility results of gram-positive bacterial isolated from patients with bloodstream infections at UoGCSH, Ethiopia.

Bacterial isolates	Antimicrobials					
	PEN	AMP	OXC	GEN	VAN	CAF
	*R* (%)	*R* (%)	*R* (%)	*R* (%)	*R* (%)	*R* (%)
CoNS (*n* = 8)	6 (100)	NA	4 (80)	3 (50)	NA	NA
*S. aureus* (*n* = 165)	120 (87.6)	NA	91 (61.1)	63 (45.3)	NA	NA
*S. viridians* (*n* = 49)	NA	NA	NA	NA	14 (31.1)	NA
*S. pneumoniae* (*n* = 11)	NA	NA	NA	NA	2 (20)	NA
*Enterococcus* spp. (*n* = 7)	NT	NA	NA	NA	4 (57.1)	0
*S. agalactiae* (*n* = 6)	NA	0	NA	NA	NT	NA
*S. pyogenes* (*n* = 5)	NA	0	NA	NA	0	NA

PEN, penicillin; AMP, ampicillin; OXC, oxacillin; GEN, gentamicin; VAN, vancomycin; CAF, chloramphenicol; R, resistant; NT, not tested; NA, not applicable.

Among Gram-negative bacterial isolates, *K. pneumoniae* showed high resistance to cefotaxime (94.4%), trimethoprim-sulfamethoxazole (91.9%), ciprofloxacin (53%), and gentamicin (66.9%). In comparison, it showed lower resistance to meropenem (30.7%) and amikacin (22.4%). *E. coli*, the second most common isolate, also demonstrated significant resistance to ampicillin (72.2%), amoxicillin-clavulanate (84.2%), ciprofloxacin (79%), and trimethoprim-sulfamethoxazole (84.4%). However, *E. coli* remained largely susceptible to meropenem (7.1%). Concerningly, *A. baumannii* and *E. cloacae* displayed considerable resistance to multiple tested antibiotics, including piperacillin-tazobactam (54.1 and 60%, respectively) and, in the case of *E. cloacae*, cefotaxime (82.4%) (Table 5).

**Table 5:** Antimicrobial susceptibility test results for Gram-negative bacterial pathogens from suspected bloodstream infections at UoGCSH, Ethiopia.

Bacterial isolates	Antimicrobials										
	AMP	AMC	PTZ	CTX	CIP	MER	SXT	GEN	AMK	CAF	TOB
	*R* (%)	*R* (%)	*R* (%)	*R* (%)	*R* (%)	*R* (%)	*R* (%)	*R* (%)	*R* (%)	*R* (%)	*R* (%)
*K. pneumoniae* (*n* = 203)	NA	NT	45 (57)	84 (94.4)	80 (53)	50 (30.7)	124 (91.9)	109 (66.9)	15 (22.4)	17 (45.6)	59 (56.5)
*E. coli* (*n* = 59)	13 (72.2)	16 (84.2)	10 (41.7)	20 (80)	28 (79)	3 (7.1)	27 (84.4)	27 (55.1)	10 (45.5)	6 (27.3)	NT
*A. baumannii* (*n* = 48)	NA	NA	20 (54.1)	NT	13 (34.2)	8 (27.6)	NA	20 (45.5)	3 (13)	NA	4 (66.7)
*E. cloacae* (*n* = 44)	NA	NA	3 (60)	14 (82.4)	8 (40)	NT	8 (57.1)	6 (35.3)	2 (33.3)	5 (22.7)	11 (68.8)
*NFGNR* (*n* = 34)	NA	NA	12 (63.2)	9 (100)	8 (42.1)	5 (21.7)	14 (60.9)	14 (63.6)	2 (15.4)	NA	1 (11.1)
*K. ozaena* (*n* = 17)	NA	4 (100)	4 (50)	6 (85.7)	5 (50)	5 (50)	7 (70)	7 (66.6)	2 (28.6)	4 (57.1)	NT
*Pseudomonas* spp. (*n* = 10)	NA	NA	NT	NA	2 (33.3)	0	NA	NA	1 (33.3)	NA	NT
*Citrobacter* spp. (*n* = 6)	NA	NA	1 (33.3)	1 (50)	2 (40)	0	4 (100)	3 (75)	0	1 (100)	NT
*K. oxytoca* (*n* = 7)	NA	1 (100)	2 (50)	NT	5 (71.4)	1 (20)	4 (100)	5 (83.3)	2 (40)	1 (33.3)	NT
*Providencia* spp. (*n* = 4)	NA	NA	1 (100)	NT	2 (100)	1 (50)	NT	NT	NT	1 (100)	NT
*P. mirabilis* (*n* = 3)	NA	1 (50)	1 (100)	NT	NT	1 (50)	1 (33.3)	1 (33.3)	1 (100)	0	NT
*K. rhinoscleromatis* (*n* = 2)	NA	NT	NT	NT	1 (50)	0	1 (100)	0	NT	0	0
*E. aerogenes* (*n* = 1)	NA	NA	NA	NT	NT	NT	1 (100)	0	0	NT	0
*P. multocida* (*n* = 1)	NA	NT	1 (100)	NT	NT	0	NT	1 (100)	0	NT	NT
*S. typhi* (*n* = 1)	NT	NT	NT	NT	1 (100)	0	NT	0	0	NT	NT
*S. arizonae* (*n* = 1)	NT	NT	1 (100)	NT	1 (100)	0	0	0	0	0	NT
*Salmonella group A* (*n* = 3)	NT	NT	NT	NT	0	0	0	0	0	NT	NT
*Serratia* spp. (*n* = 1)	NA	NA	NA	0	1 (100)	0	0	0	1 (100)	NT	1 (100)
*N. meningitidis* (*n* = 3)	NA	NA	NA	NT	2 (50)	1 (50)	2 (66.7)	NA	NA	1 (100)	NA

AMP, ampicillin; AMC, amoxicillin-clavulanate; PTZ, piperacillin_tazobactam; CTX, cefotaxime; CIP, ciprofloxacin; MER, meropenem; SXT, trimethoprim-sulfamethoxazole; GEN, gentamicin; AMK, amikacin; CAF, chloramphenicol; TOB, tobramycine; R, resistant; NT, not tested; NA, not applicable.

### Multidrug-resistant bacterial isolates

Multidrug resistance was a common characteristic observed across the bacterial isolates, irrespective of their Gram-staining properties. Of the 701 bacterial isolates analyzed, 89.9% (630) exhibited resistance to at least one antimicrobial agent included in the susceptibility tests. Moreover, 60.9% (427) of these isolates displayed resistance to three or more distinct classes of antimicrobial agents. Specifically, *K. oxytoca* (100%), *K. pneumoniae* (97.5%), *E. coli* (96.6%), *Citrobacter* spp. (85.7%), and *Acinetobacter baumannii* (81.6%) demonstrated high levels of multidrug resistance, consistent with the finding of resistance to multiple antimicrobial categories (Table 6).

**Table 6:** Multidrug-resistant bacterial isolates from patients with bloodstream infections at UoGCSH, 2024.

Bacterial isolates	Degree of microbial resistance					Total MDR isolates ≥ R3
	R0	R1	R2	R3	R4	
Gram negative bacteria						
*K. pneumoniae* (*n* = 203)	0	1 (0.5)	4 (2.0)	34 (16.7)	61 (30.0)	198 (97.5)
*E. coli* (*n* = 59)	1 (1.7)	0	1 (1.7)	11 (18.6)	16 (27.1)	57 (96.6)
*A. baumannii* (*n* = 49)	0	1 (2.0)	7 (14.3)	10 (20.4)	28 (57.1)	40 (81.6)
*E. cloacae* (*n* = 48)	0	3 (6.3)	15 (31.3)	6 (12.5)	7 (14.6)	26 (54.2)
*NFGNR* (*n* = 44)	0	0	3 (6.8)	12 (27.3)	14 (31.8)	31 (70.5)
*K. ozaenae* (*n* = 34)	0	2 (5.9)	1 (2.9)	3 (8.8)	3 (8.8)	14 (41.2)
*Pseudomonas* spp. (*n* = 10)	1 (10)	1 (10)	4 (40)	3 (30)	1 (10)	4 (40)
*K. oxytoca* (*n* = 7)	0	0	0	2 (28.6)	1 (14.3)	7 (100.0)
*Citrobacter* spp. (*n* = 7)	0	0	0	0	2 (28.6)	6 (85.7)
*Providencia* spp. (*n* = 5)	2 (40)	0	0	0	1 (20)	2 (40)
*N. meningitidis* (*n* = 5)	3 (50.0)	1 (16.7)	1 (16.7)	0	0	0
*S. pyogenes* (*n* = 5)	5 (100)	0	0	0	0	0
*P. mirabilis* (*n* = 4)	0	0	0	1 (25)	1 (25)	3 (75)
*Salmonella* group A (*n* = 3)	0	2 (66.7)	1 (33.3)	0	0	0
*K. rhinoscleromatis* (*n* = 2)	0	0	0	0	2 (100)	2 (100)
*E. aerogenes* (*n* = 1)	0	0	0	1 (100)	0	1 (100)
*P. multocida* (*n* = 1)	0	0	0	1 (100)	0	1 (100)
*S. arizonae* (*n* = 1)	0	0	0	1 (100)	0	1 (100)
*S. typhi* (*n* = 1)	0	0	0	1 (100)	0	1 (100)
*Seratia* spp. (*n* = 1)	0	0	0	0	0	1 (100)
Total	12 (1.7)	11 (1.6)	29 (5.3)	86 (12.3)	137 (19.5)	395 (56.3)
Gram positive bacteria						
*S. aureus* (*n* = 165)	22 (13.3)	66 (40.0)	49 (29.7)	10 (6.1)	13 (7.9)	28 (17.0)
*S. viridians* (*n* = 49)	25 (51.0)	13 (26.5)	9 (18.4)	2 (4.1)	0	2 (4.1)
*S. pneumoniae* (*n* = 11)	8 (72.7)	3 (27.3)	0	0	0	0
*CoNS* (*n* = 10)	1 (10)	4 (40)	2 (20)	1 (10)	0	1 (10)
*Enterococcus* spp. (*n* = 8)	0	3 (37.5)	3 (37.5)	1 (12.5)	0	1 (12.5)
*S. agalactiaee* (*n* = 6)	3 (50.0)	2 (33.3)	1 (16.7)	0	0	0
Total	59 (8.4)	91 (13.0)	63 (9.1)	14 (2.0)	13 (1.9)	32 (4.6)
Overall total	71 (10.1)	102 (14.6)	101 (14.4)	100 (14.3)	150 (21.4)	427 (60.9)

R0, No resistance to antibiotics; R1, Resistant to one type of antimicrobial agent; R2, Resistant to two different types of antimicrobial agents; R3, Resistant to three different types of antimicrobial agents; R4, Resistant to four or more different types of antimicrobial agents.

### Factors associated with bacterial bloodstream infections

The bivariable and multivariable logistic regression analyses revealed significant associations between specific demographic and clinical factors and BSI. The age groups of study participants were found to have a substantial impact, with study participants less than 1 month old (AOR = 6.8, 95, 95% CI = 2.41–19.09, *p* < 0.001) and those infants and toddlers aged 1 month to less than 3 years old (AOR = 2.0, 95% CI = 1.54–2.64, p < 0.001) showed significant association with BSIs. Furthermore, patients who visited the emergency OPD (AOR = 3.2, 95% CI = 1.50–6.74, *p* = 0.003), those admitted to the oncology ward (AOR = 3.0, 95% CI = 1.21–7.17, *p* = 0.017), and those in the surgical ward (AOR = 3.3, 95% CI = 1.27–8.43, *p* = 0.014) were identified as being at higher risk for developing BSIs (Table 7).

**Table 7:** Multivariable logistic regression analysis results in bacterial culture-positive and potential associated factors at UoGCSH, 2024.

Variables	Category	Culture result		AOR (95% CI)	*p*-value
		Negative *n* (%)	Positive *n* (%)		
Gender	Male	2,323 (49.1)	424 (9.0)	1.1 (0.95–1.34)	0.166
	Female	1,703 (36.0)	277 (5.9)	1	
Patient category	Neonate	959 (20.3)	285 (6.0)	0.5 (0.06–4.74)	0.573
	Pediatric	1,537 (32.5)	284 (6.0)	0.8 (0.31–2.0)	0.612
	Adult	1,530 (32.4)	132 (2.8)	1	
Age	Below 1 month	967 (20.5)	292 (6.2)	6.8 (2.41–19.09)	<0.001*
	1 month to <3 years	730 (15.4)	179 (3.8)	2.0 (1.54–2.64)	<0.001*
	3 years and above	2,329 (49.3)	230 (4.9)	1	
Patient’s clinical setting	Medical OPD	648 (13.7)	53 (1.1)	1	
	Neonatal ICU	961 (20.3)	285 (6.0)	1.0 (0.11–8.60)	0.986
	ETAT	657 (13.9)	124 (2.6)	2.0 (0.76–5.53)	0.159
	Adult inpatient	587 (12.4)	37 (0.8)	0.8 (0.54–1.30)	0.420
	Pediatric ICU	462 (9.8)	100 (2.1)	2.4 (0.88–6.61)	0.086
	Pediatric inpatient	365 (7.7)	51 (1.1)	1.6 (0.57–4.42)	0.376
	Medical ICU	176 (3.7)	16 (0.3)	1.2 (0.66–2.09)	0.616
	Emergency OPD	41 (0.87)	10 (0.21)	3.2 (1.50–6.74)	0.003*
	Gynecology ward	13 (0.3)	3 (0.1)	3.2 (0.88–11.76)	0.076
	Oncology ward	60 (1.3)	13 (0.3)	3.0 (1.21–7.17)	0.017*
	Surgical OPD	33 (0.7)	3 (0.1)	1.2 (0.34–3.94)	0.819
	Surgical ward	23 (0.5)	6 (0.1)	3.3 (1.27–8.43)	0.014*
Year	2019	929 (19.7)	181 (3.8)	1	
	2020	1,405 (29.7)	206 (4.4)	1.2 (0.9–1.49)	0.156
	2021	1,692 (35.8)	313 (6.6)	0.3 (0.75–1.10)	0.904
Fever	Yes	3,552 (75.1)	592 (12.5)	0.9 (0.72–1.22)	0.641
	No	474 (10.0)	109 (2.3)	1	
Fever during the last 48 h	Yes	109 (2.3)	9 (0.2)	1.1 (0.82–1.38)	0.641
	No	3,917 (82.9)	692 (14.6)	1	
Increased respiratory rate	Yes	941 (19.9)	174 (3.7)	1.1 (0.93–1.37)	0.216
	No	3,085 (65.3)	527 (11.2)	1	
Hypotension	Yes	111 (2.4)	8 (0.2)	0.5 (0.26–1.13)	0.102
	No	3,915 (82.8)	693 (14.7)	1	
Antibiotic therapy within 2 weeks before diagnosis	Yes	1,428 (30.2)	252 (5.3)	1	
	No/unknown	2,598 (55.0)	449 (9.5)	1.0 (0.83–1.17)	0.838

* ETAT, emergency triage assessment and treatment; *statistically significant association.

## Discussion

Bloodstream infections are a major public health concern due to the growing threat of AMR globally. The prevalence and resistance profiles of bacterial pathogens isolated from BSIs in Ethiopia showed significant regional variations. The reported prevalence of bacterial pathogens in suspected BSI cases in the current study (14.8%) demonstrates both consistencies and discrepancies when compared to other studies. The alignment with findings from Nepal (12.6 and 15%) (Ansari et al., 2015; Manandhar et al., 2021), India (14.2%) (Banik et al., 2018), and Italy (16.0%) (Biagio et al., 2020) suggests a degree of global comparability in BSI prevalence within certain settings. However, the significant divergence from previously reported rates within Ethiopia itself (5.4–11.3%) (Birru et al., 2021; Ameya et al., 2020; Tufa et al., 2022), as well as Tanzania (3.2%) (Coline et al., 2015) and Iran (9.6%) (Alka et al., 2019) highlight the importance of considering contextual factors. These internal Ethiopian variations likely stem from differences in study design, including sample size and patient selection criteria. Studies conducted in specific high-risk populations or during periods with heightened infection rates might naturally yield higher prevalence. Furthermore, temporal variations in healthcare practices, including infection control measures and antibiotic usage, could contribute to these fluctuations. The lower prevalence reported in Tanzania and Iran may reflect differences in diagnostic capabilities, healthcare infrastructure, and the overall burden of infectious diseases within those specific regions.

Conversely, the higher prevalence rates documented in other Ethiopian studies (18.2–31.9%) (Belew et al., 2023; Legese et al., 2022; Sorsa et al., 2019; Wasihun et al., 2015; Abebaw et al., 2018; Abebe et al., 2021; Dagnew et al., 2013; Muleta et al., 2022; Beshah et al., 2022) and across various low and middle-income countries including India (31.2%) (Nikita et al., 2016), Cameroon (16%) (Yimtchi et al., 2023), and Nigeria (19.2%) (Oyekale et al., 2022), and even reaching alarmingly high levels in specific settings within Tanzania (38.9%) (Kayange et al., 2010), Nigeria (43.5%) (Oliemen et al., 2015), Ethiopia (50.6%) (Tsegaye et al., 2021), Ghana (51.4%) (John et al., 2019), paint a concerning picture. These high rates are likely a consequence of several factors. Varying diagnostic methodologies play a crucial role. Differences in blood culture techniques, including the volume of blood cultured and automated systems versus manual methods, can significantly impact pathogen detection rates. Moreover, the intensity and effectiveness of infection control initiatives within healthcare facilities directly affect BSI prevalence.

The distribution and frequency of bacterial pathogens exhibited significant variation across different geographical and clinical settings (Sorsa et al., 2019; Alka et al., 2019; Abayneh et al., 2021; Fenta et al., 2022; Gebrehiwot et al., 2012; Geyesus et al., 2017; Wen et al., 2021). In the present study, Gram-negative bacteria were the most prevalent (63.5%), consistent with findings from low and middle-income countries (60%) (Wen et al., 2021), India (60.37%) (Banik et al., 2018), and Ethiopia (60%) (Tsegaye et al., 2021). However, lower prevalence rates were reported in Iran (55.4%) (Maham et al., 2018), Nepal (52.3 and 50.5%) (Ansari et al., 2014; Parajuli et al., 2017), Nigeria (53.6%) (Oliemen et al., 2015), and Ethiopia (54.5 and 55.6%) (Belew et al., 2023; Beshah et al., 2022). The current prevalence is lower than that reported in Cameroon (75%) (Yimtchi et al., 2023) and Ghana (72.0%) (Edna et al., 2022). Among Gram-positive bacteria, *S. aureus* (22.0%) was the most frequently isolated organism, consistent with findings from Ethiopia (23.9 and 26.7%) (Belew et al., 2023; Dagnew et al., 2013; Muleta et al., 2022; Fenta et al., 2022) and Iran (20.6%) (Alka et al., 2019). However, this result is higher than those reported in Nepal (14.6%) (Ansari et al., 2015). Conversely, the prevalence from the present study is lower than that reported in Ethiopia (29.3 to 47.9%) (Birru et al., 2021; Ameya et al., 2020; Wasihun et al., 2015; Gebrehiwot et al., 2012; Geyesus et al., 2017), Nigeria (51.5%) (Oliemen et al., 2015), and India (52%) (Nikita et al., 2016). Among Gram-negative isolates, *K. pneumoniae* (27.1%) was the predominant species, exceeding rates observed in Ethiopia (12.9 and 17.6%) (Dagnew et al., 2013; Beshah et al., 2022) and Ghana (13.6%) (Edna et al., 2022). However, our finding is lower than reports from Nepal (34%) (Manandhar et al., 2021) and Ethiopia (26.1%) (Legese et al., 2022).

Antimicrobial-resistant bacterial pathogens are increasingly emerging in response to commonly used antibiotics. In the current study, 87.6 and 61.1% of *S. aureus* isolates were resistant to penicillin and oxacillin, respectively. The emergence of methicillin (oxacillin) resistant *S. aureus* (MRSA) is a major concern, especially in low-income countries like Ethiopia, where patient management facilities are limited. Nevertheless, the current finding of lower resistance (61.1% to penicillin) contrasts with the 79% reported in Malawi (Patrick et al., 2017). Furthermore, our study revealed that *S. pyogenes* exhibited 100% susceptibility to vancomycin. When it comes to Gram-negative bacterial isolates, varying levels of susceptibility to antimicrobial agents were observed. Specifically, 61.1% of *K. pneumoniae* isolates were resistant to trimethoprim-sulfamethoxazole, while 53.7% were resistant to meropenem. The emergence of carbapenem-resistant *K. pneumoniae* strains is particularly concerning due to their ability to confer resistance to a wide range of antibiotics. A high (63.6%) rate of carbapenem-resistant *K. pneumoniae* strains was found in Spain (Xu et al., 2018). Similarly, 41.7% of *A. baumannii* isolates showed resistance to piperacillin/tazobactam and gentamicin and 16.7 to meropenem. Unfortunately, all *K. ozaena* isolates demonstrated 100% resistance to gentamicin and trimethoprim-sulfamethoxazole and 71% to ciprofloxacin and meropenem.

The high prevalence of MDR (60.9%) isolates in this study aligns with the global trend of escalating AMR and is particularly worrisome. The likely contributors to this high prevalence include suboptimal antimicrobial utilization, such as overuse and misuse of antibiotics (Christaki et al., 2019; Munita and Arias, 2016; Belachew et al., 2021), which exert significant selective pressure (Christaki et al., 2019). The absence of stringent antimicrobial stewardship programs further exacerbates this issue (Hawkey et al., 2018; Langendonk et al., 2021).

The analysis reveals that study participants under 1 month of age are 6.8 times more likely to develop bloodstream infections compared to participants aged 3 years and older. Similarly, children aged between 1 month and less than 3 years also face a significantly increased risk, with a twofold higher likelihood of developing BSIs compared to their older peers. This might be due to their underdeveloped immune systems (Zaoutis et al., 2005; Kim et al., 2022; Kłos and Wójkowska-Mach, 2019; Borghesi et al., 2020), pathogen exposure (Luthander et al., 2020; Modler et al., 2023). Similarly, the higher susceptibility among patients in the emergency department, oncology ward, and surgical wards is understandable given the nature of their conditions and the intensity of medical interventions they undergo. Traumatic injuries, invasive procedures, and immunocompromised states all contribute to an elevated risk of BSI in these settings. The fast-paced environment of emergency care can also lead to unintentional breaches in infection control protocols (Yang et al., 2018; Dumnui et al., 2022).

### Study limitations

The study’s limitations include its single-center design, potentially restricting the generalizability of findings. The lack of molecular typing also limits insights into genetic mechanisms driving resistance and pathogen evolution. Future multicenter studies incorporating molecular epidemiology are essential to provide a more comprehensive understanding of BSI dynamics.

## Conclusion

The overall prevalence of bacterial isolates was high, with Gram-negative bacteria, particularly *K. pneumoniae*, being the most frequently identified pathogens, followed by the Gram-positive bacterium *S. aureus*. Alarmingly, significant antimicrobial resistance was observed with concerning levels of MDR. Moreover, the study identified significant associations between BSI and age groups (infants under 1 month and children aged 1 month to less than 3 years) and presentation in specific clinical settings, such as the emergency OPD, oncology ward, and surgical ward.

### Recommendations

- To improve patient safety, the hospital should implement and enhance its antimicrobial stewardship program and strengthen its infection prevention and control measures.
- The Federal Ministry of Health needs to develop and implement a national strategy to combat AMR while also investing in strengthening laboratory capacity for microbiology diagnostics.
- For effective local implementation, the Regional Health Bureau should facilitate and support national AMR strategies and establish a robust surveillance system for bloodstream infections and antimicrobial resistance within the region.

## Data Availability

The original contributions presented in the study are included in the article/supplementary material, further inquiries can be directed to the corresponding author.
